# Fertility awareness and knowledge among Indian women attending an infertility clinic: a cross-sectional study

**DOI:** 10.1186/s12905-018-0669-y

**Published:** 2018-10-29

**Authors:** Reeta Mahey, Monica Gupta, Shobha Kandpal, Neena Malhotra, Perumal Vanamail, Neeta Singh, Alka Kriplani

**Affiliations:** 1 Department of Obstetrics & Gynecology, All India Institute of Medical Sciences, New Delhi, India; 2 Department of Obstetrics & Gynecology, Fellow Reproductive Medicine, All India Institute of Medical Sciences, Room no3076, Third Floor, Teaching Block, Ansari Nagar-, New Delhi, 110029 India; 3 Department of Obstetrics & Gynecology, Medical Social Service Officer, All India Institute of Medical Sciences, New Delhi, India

**Keywords:** Fertility, Awareness, Infertility, Knowledge, Parenthood

## Abstract

**Background:**

To evaluate fertility knowledge and awareness among infertile women attending an Indian assisted fertility clinic and their understanding of the menstrual cycle, how age affects fertility and need for assisted fertility treatment.

**Methods:**

A cross sectional study was conducted including 205 women seeking fertility treatment at an assisted reproductive unit between March 2017 to August 2017. Patients were interviewed with the help of structured questionnaire by a fertility counsellor. The previous studies were reviewed and a questionnaire was made according to our patient profile and sociodemographic characteristics. Knowledge and awareness was stratified according to socioeconomic status (SES).

**Results:**

Most women (59%) were aged between 20 to 30 years indicating concern about their fertility and need for evaluation. More than half (63%) women were from the middle socio-economic strata. Knowledge about fertility and reproduction was low: 85% were not aware of the ovulatory period in the menstrual cycle, only 8% considered age more than 35 years as the most significant risk factor for infertility and most were unaware of when to seek treatment for infertility after trying for pregnancy. Less than half of women understood the need for assisted fertility treatment and donor oocytes in advanced age.

**Conclusions:**

Most Indian women across different SES are unaware of the effect of age on fertility. Targeted educational interventions are needed to improve knowledge regarding ideal age of fertility, factors affecting fertility potential and fertility options available for sub-fertile couples. Fertility counselling and information should be provided to young people at every contact with health care professionals.

## Background

Parenthood is a dream of many couples, however they may not plan a pregnancy keeping advancing age and issues pertaining to infertility in mind [1]. Both unplanned pregnancy and infertility occur commonly [2]. Traditionally fertility awareness was considered to be knowledge of female anatomy and physiology and its application to family planning [3]. However, as age at first conception is increasing globally, the epidemic of infertility looms large. The global trend in delaying parenthood is being attributed to a number of factors, primarily, pursuit of higher education and career goals, desire for a stable job and delay in finding a suitable partner. In UK in 2013, average age of mothers at first birth was 28.3 years versus 26.6 years in 2001 and approximately half of all live births (51%) were to mothers aged 30 and over [4]. It has been observed that couples have a basic knowledge of factors affecting fertility, but remain unaware of the impact that advancing age has on a women’s fertility [3, 5]. It is well established that female fertility declines after age of 30 and more rapidly once women turn 35 years [5, 6]. The advent of assisted reproductive technique (ART) and its widespread availability has helped many couples realize their dream of parenthood [7].

A major factor responsible for delayed child bearing and increasing incidence of subfertility is lack of awareness about fertility potential. Decisions about whether, when and how to conceive, should be a matter of individual or couples’ choice. However, an accurate understanding of reproductive facts is essential for informed fertility decision making. Currently awareness about fertility is low worldwide [2, 5].

Until recent years in India, there was a trend of early marriage and having a first child at less than 25 years, but now due to socioeconomic development and greater interest in education, job and financial settlement there is a delay in parenthood. Most of the women now plan their first pregnancy after 30 years of age. As in other developing countries, in India, having children is the social norm while childlessness is socially stigmatized [8]. Infertility has a profound effect on the psychological and social well-being of women much more than men [9]. In recent years the fertility rate has declined in virtually all regions of the world and a recent report by United Nations has stated that the fertility rate of Indians has decreased by half in last 40 years [10].

Although the incidence of infertility is on rise, no study till date has evaluated fertility awareness among Indian women. Determining the level of knowledge and awareness of fertility practices among Indian women has important public health implications. Targeted fertility education and public enlightenment programmes may help in reducing the number of women experiencing age related infertility and also enable timely referral for assisted fertility treatment. The present study was conducted to evaluate knowledge of women attending infertility clinic regarding factors affecting fertility and availability of advanced infertility treatment options.

## Methods

This was a cross sectional study conducted from March 2017 to August 2017 at infertility clinic of All India Institute of Medical Sciences, New Delhi, India.

### Study population

**Fig. 1:**
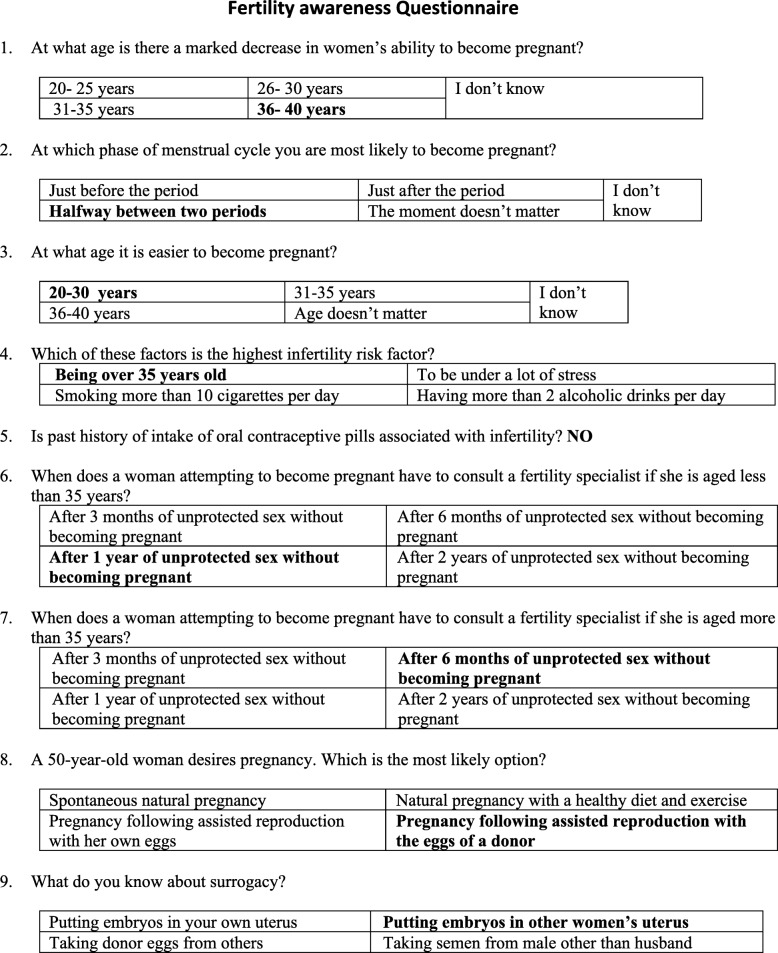
Nine Item Questionnaire with possible correct answers highlighted

**Fig. 2:**
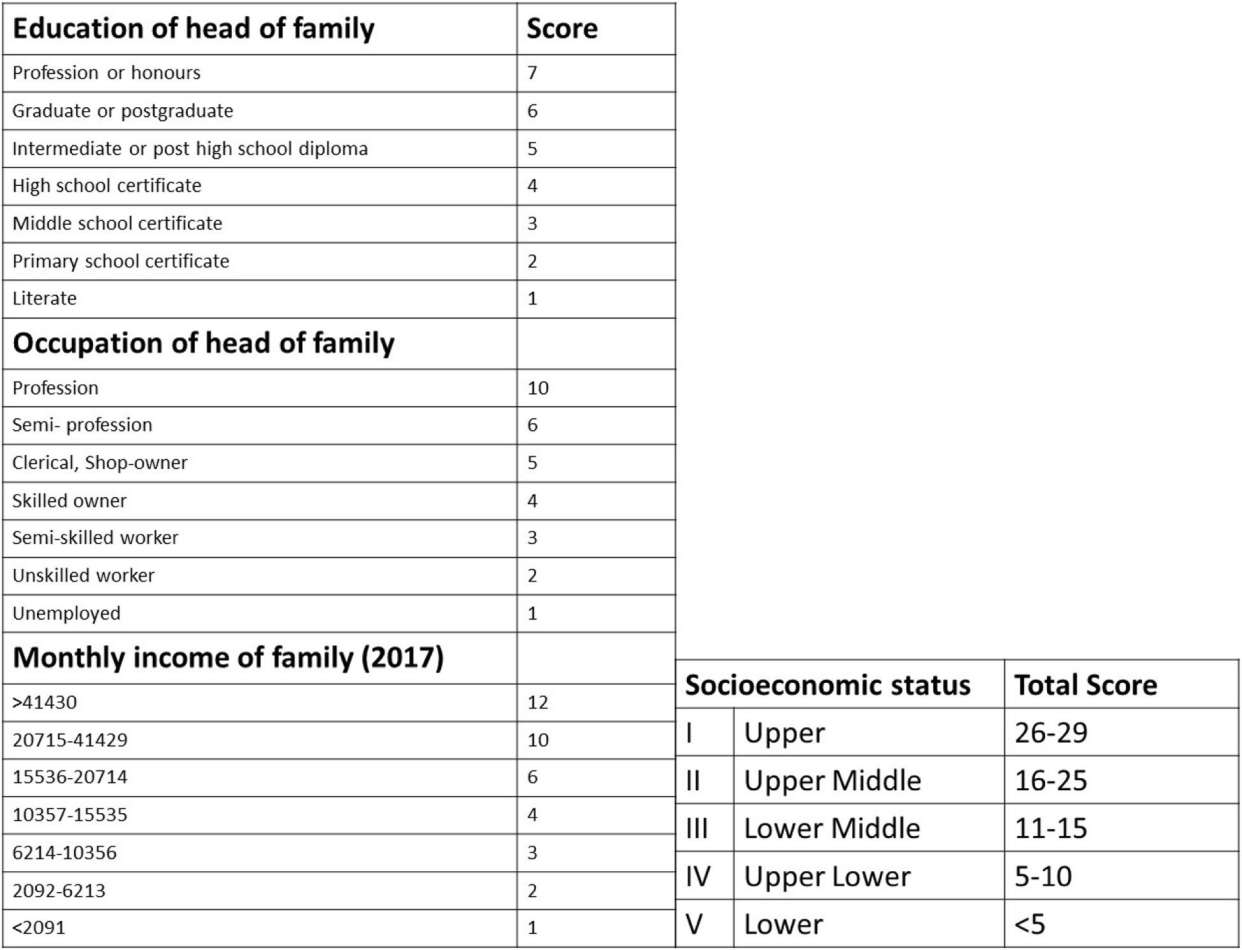
Modified Kuppuswamy Socioeconomic scale

A total of 205 women attending infertility clinic participated in the study. The inclusion criteria were women of age group 21 to 44 years who either directly consulted the infertility clinic or were referred form general OBGYN clinic and were trying for conception for at least 6 months. Women were invited to participate in the study at the initial consultation and those who voluntarily gave written consent were interviewed by a fertility counsellor. The interview consisted of a nine item questionnaire which was designed after reviewing previous papers on fertility awareness and modified according to patient population and level of understanding in an Indian set up [5, 11–13]. The patients had to identify the correct answers from the options given after each question. There were questions regarding the age related decline in fertility, the fertile period in the menstrual cycle, relation of oral contraceptive pill intake with fertility, the duration after which to consult a fertility specialist after trying for pregnancy, fertility options for women in advanced age (> 40 years) and those with non functional uterus. Figure 1 depicts the questions and possible correct answers in bold. Baseline demographic parameters were recorded and socio-economic status (SES) calculated according to Modified Kuppuswamy scale. The modified Kuppuswamy scale consisted of three parameters – education and occupation of head of family and total family income. According to the score obtained in each of these three parameters the participants were grouped in five SES classes as shown in Fig. 2 [14]. The correct answers were stratified according to SES. Ethical approval was obtained from the Institute Ethics committee before starting the study.

### Statistical analysis

All statistical analysis was carried out using SPSS, IBM version 19 (Armonk, NY: IBM Corp). Descriptive statistics were used for quantitative variables and were expressed as mean, standard deviation or frequencies, and percentages. The percentage of participants answering correctly was stratified according to SES scale. For comparing categorical data, Pearson’s chi square test or Fishers exact test was carried out as appropriate. For all statistical tests *P* < 0.05 was considered to be statistically significant.

## Results

### Demographic characteristics

The baseline demographic characteristics of the study population are presented in Table 1. A total of 205 women were interviewed. More than half of women (121/205; 59%) belonged to the age group of 20 to 30 years. Three fourths of the patients had good marital relations that is the couples were compatible and seldom had issues pertaining to the stress of infertility. When the study population was stratified into various SES according to modified Kuppuswamy scale, the majority of participants (130/205; 63%) belonged to the middle SES. Only 29 (14.1%) belonged to the upper lower category and there were no subjects in lower category. The frequency of participants who identified the correct answers among various SES categories is as shown in Table 2.

**Table 1:** Demographic profile of study population

Baseline characteristics	Mean ± SD (*n* = 205)	
Age (Wife) years	29.96 ± 4.67	
Age (Husband) years	33.97 ± 5	
Marriage duration (years)	7.88 ± 4.52	
Infertility duration (years)	6.10 ± 4.24	
Frequency (%) (*n* = 205)		
Age group (years)	20–30	121 (59)
	30–40	81 (39.5)
	>40	3 (1.5)
Socioeconomic status^a^	Upper	46 (22.4)
	Upper Middle	77 (37.6)
	Lower Middle	53 (25.9)
	Upper lower	29 (14.1)
Infertility	Primary	153 (74.6)
	Secondary	52 (25.4)
Marital relations	Good	155 (75.6)
	Bad	50 (24.4)

^a^Socioeconomic status was calculated according to modified Kuppuswamy scale [11]

**Table 2:** Frequency of correct answers overall and according to Kuppuswamy socioeconomic scale

Questions (Correct answers)	Overall (*n* = 205)	Upper (*n* = 46)	Upper middle (*n* = 77)	Lower middle (*n* = 53)	Upper lower (*n* = 29)	*P*
After which age it becomes difficult to achieve pregnancy? (>35 years)	53 (26%)	17 (37%)	21 (27.3%)	10 (18.9%)	5 (17.2%)	0.13
Which phase of menstrual cycle you are more likely to get pregnant? (mid-cycle)	31 (15.1%)	6 (13%)	17 (22.1%)	5 (9.4%)	3 (10.3%)	0.17
At what age it is easier to become pregnant? (20–30 years)	175 (85.4%)	41 (89.2%)^a^	72 (93.5%)^a^	40 (75.5%)	22 (75.9%)	0.04
Which of these is highest risk factor for infertility? (>35 years)	16 (7.8%)	3 (6.5%)	9 (11.7%)	4 (7.5%)	0	0.24
Is past history of intake of COC, associated with infertility? (No)	6 (2.9%)	2 (4.3%)	2 (2.6%)	0	2 (6.9%)	0.3
When does a woman attempting to become pregnant have to consult a fertility specialist if she is aged less than 35 years? (1 year)	79 (38.5%)	19 (41.3%)	30 (39%)	23 (43.4%)	7 (24%)	0.35
When does a woman attempting to become pregnant have to consult a fertility specialist if she is aged more than 35 years? (6 months)	37 (18%)	10 (21%)	12 (15.6%)	10 (18.9%)	5 (17.2%)	0.85
A 50-year-old woman desires pregnancy. Which is the most likely option? (Donor oocyte IVF)	54 (26.3%)	18 (39.1%)	21 (27.3%)	11 (20.8%)	4 (13.8%)	0.06
What do you know about surrogacy? (Putting embryos in other women’s uterus)	88 (42.9%)	26 (56.5%)^a^	40 (51.9%)^a^	15 (28.3%)	7 (24.1%)	0.002

^a^Pearson-Chi squared test; *p* value < 0.05 was considered statistically significant

When we assessed the response in relation to age related decline in fertility, it was found that most participants (> 85%) were aware that young women are more fertile and it is easier to achieve pregnancy between 20 to 30 years. This awareness was demonstrated across all socioeconomic strata but significantly better in upper and upper middle class. However, only 26% (53/205) participants could correctly identify the critical age threshold of 35 years after which it becomes difficult to achieve pregnancy. On enquiry regarding the ovulatory period, it was found that 85% participants failed to correctly identify the mid cycle as the most likely phase to achieve pregnancy. This knowledge was consistently poor among all SES classes. Almost 97% (193/205) participants associated past intake of combined oral contraceptive pills with infertility. This belief was similarly demonstrated in all classes of participants. Sixty to 80 % of participants were not aware of the duration after which to consult a fertility specialist if they have been trying for pregnancy and are unable to achieve the same. Only 26% women were aware of the need of assisted reproduction and donor oocyte at the age of 50 years. On subgroup analysis according to SES, women in the upper SES had better knowledge (39% correctly answered) regarding need of ART and the option of donor oocyte in advanced age than in other categories. Similarly, knowledge regarding surrogacy was also higher among women belonging to upper SES category than in the lower category (more than 50% in upper SES correctly answered versus 24% in lower SES).

## Discussion

There is a worldwide increase in age at first conception and decline in fertility rate [15]. Infertility is a major public health concern and globally affects 1 in 6 couples, with more prevalence in developing countries [16]. In India, parenthood is considered a true indicator of a happy married life. Like other developing countries, infertility is a taboo topic in India and a lot of social stigma is associated with childlessness [8]. Failure to conceive is not only very depressing for couples but may also affect sexual life and relationship with friends and family. Worldwide studies have shown that people are unaware of biological aspects of conception, have poor knowledge about the most fertile period in the menstrual cycle, the chances of getting pregnant in one cycle and about the steep decline in fertility potential after age of 34–35 years [5, 17, 18]. Also both men and women lack knowledge about the effects of smoking, alcohol, job stress and other life style factors on fertility potential.

Our study population consisted of women attending infertility clinic who either consulted directly or were referred from general OBGYN clinic. These women are supposed to have more knowledge about infertility than general population. Although majority of participants (85%) were aware that younger age group (20 to 30 years) is more fertile and fertility declines with age however they were unable to identify the critical threshold of 35 years after which fertility rapidly declines. The participants in the upper and upper middle SES class had better knowledge compared to the lower SES classes. Similar findings were also reported in a study of fertility awareness among oocyte donors in Spain in which the majority of participants (75%) overestimated the age of decline in fertility [12]. Another important highlight of our study was that the fertile window in the menstrual cycle was missed by approximately 85% participants. Similar findings were reported by Hampton et al. in their study on fertility awareness. They reported that although 88.1% participants believed that they were aware of the fertile period in the menstrual cycle, but only 12.7% could accurately identify the fertile days [19]. In our study even patients belonging to the upper and middle SES class had lack of knowledge about the fertile period. Hampton et al. also stated that there was no association between high fertility awareness and socioeconomic status. This is in contrast to the assumption that higher education and higher societal class indicates greater fertility knowledge as shown in other studies [2]. The reason behind this might be the lack of fertility education during the formative years of school and college life in our country.

Our study also highlights another important finding regarding association of oral contraceptive pills and infertility. Nearly 97% of women believed that past history of pill intake was associated with infertility. People often incorrectly attribute the age related decline in fertility or other unexplained cause of infertility to contraceptive pill use. In a study conducted in African women between 37 and 62% of women believed that contraceptives could harm the womb [20]. Implementation of contraceptive education at primary level is essential to dispel such myths.

The majority of respondents (74%) in our study believed that it was possible to achieve pregnancy at 50 years either naturally or with the help of ART with self oocytes. This over optimism and belief that it is possible to have children naturally until the onset of menopause has also been shown in previous studies [5, 21].

Knowledge of the need of donor oocytes and assisted reproduction in women of advanced age was better in upper and upper middle SES classes. Patients in the upper SES were significantly more aware about surrogacy and its implications. Bunting et al. have shown in their study that variation in fertility knowledge is mainly related to socio-demographic factors like education, employment and development index [2]. Similarly, we have also found that participants from the upper and upper middle SES were more knowledgeable especially regarding ART treatment options due to better education and more access to media and internet.

Most of the studies on fertility awareness have been conducted in women only. Since, men play a major role in childbearing decisions and timing, it is important to assess their level of understanding and knowledge of factors affecting fertility [5].

Taking into account low fertility knowledge among men and women, British Fertility Society has started Fertility Education Initiative (FEI) in association with Royal College of Obstetricians and Gynaecologists and few other stakeholders to help all women and men so that they are able to make informed choices about their fertility planning [15]. The FEI aims to increase fertility awareness by developing a number of interactive tools for teachers, young people and health professionals which will help young people to make well informed choices regarding reproduction and family planning [15].

Taking into account the increasing prevalence of infertility and referrals to ART clinics, accurate fertility information should be provided widely to the masses through awareness programmes, media and at the time of contact with healthcare professionals. Programmes similar to FEI can be started in India which can be integrated with education curriculum to increase fertility awareness. As it is with other areas of health, one should be empowered with sufficient knowledge about fertility potential and planning. It is mandatory to have methods and tools available to spread fertility awareness among all potential couples, college students and carrier settlers so that they will not face the reality of infertility in the twenty first century.

Our study is the first of its kind to be conducted in India and has highlighted some very important issues of both national and international significance. We assessed the SES of the study population which has not only provided a better understanding of variation in knowledge among different SES categories but also indicated that higher socioeconomic status does not necessarily mean a better awareness of fertility knowledge. However, there are some biases in our study. We interviewed women from the fertility clinic of a tertiary care centre and they may not represent the general population. Prior consultation for infertility has been found to improve knowledge in some areas of infertility [2].

## Conclusion

The present study has identified significant important gaps in women’s knowledge and awareness regarding fertility practices. These include poor knowledge of factors affecting fertility especially age related decline in fertility, fertile period in the menstrual cycle and myths regarding oral pill intake and risk of infertility. The study also concluded that higher socioeconomic status and better education does not translate into improved knowledge and awareness of factors affecting fertility. Better accessibility of higher SES classes to media and internet may have enhanced their knowledge regarding advanced fertility treatment options, but they still lacked the basic knowledge of fertility affecting factors. Hence, there is a need of generalized education of masses and targeted interventions at primary level. The most important information that needs to be transmitted to young infertile couples is when it would be biologically too late to achieve childbearing and availability of other ART facilities at late age. Future research should focus on assessing the knowledge of infertile males and also professionals involved in primary health care.

## Abbreviations

ART: Assisted Reproductive Technique
FEI: Fertility Education Initiative
SES: Socio Economic Status
